# Resource-efficient use of industrial residue for multifunctional cacao nanofibers

**DOI:** 10.3389/fbioe.2026.1916485

**Published:** 2026-10-05

**Authors:** Linn Berglund, Io Antonopoulou, Luísa R. Völtz, Pooja Dixit, Carlos Martín, Ola Sundman, Jenny Berg, Kristiina Oksman

**Affiliations:** 1 Wood and Bionanocomposites, Division of Materials Science, Department of Engineering Sciences and Mathematics, Luleå University of Technology, Luleå, Sweden; 2 Biochemical Process Engineering, Division of Chemical Engineering, Department of Civil, Environmental and Natural Resources Engineering, Luleå University of Technology, Luleå, Sweden; 3 Department of Chemistry, Umeå University, Umeå, Sweden; 4 Department of Biotechnology, University of Inland Norway, Hamar, Norway; 5 Nordic Chocolate AB, Umeå, Sweden

**Keywords:** bioactive compounds, cacao husk, fibrillation, intrinsic composition, nanocellulose, redispersion

## Abstract

This study evaluates cacao residue from chocolate production for resource-efficient production of multifunctional nanofibers. The underlying hypothesis is that preserving non-cellulosic constituents during processing can enable resource-efficient nanofiber production while contributing unique functionalities. Cacao residue yielded 30% after bleaching, with the main components being fat and holocellulose. Fibrillation produced cacao nanofibers at an energy demand of 3.9 kWh/kg, compared with the reference, a commercial wood pulp, with 8.2 kWh/kg after the same processing time. The cacao nanofibers displayed bioactivity, hydrophobic behavior, and excellent redispersibility not observed for the reference material, which has cellulose as the main component. The bioactivity of cacao nanofibers included strong antioxidant, mild antibacterial, and moderate anti-collagenase activity. Utilization of the natural composition offers a resource-efficient approach to unlock the potential of cacao residue into specific multifunctional nanofibers with potential for further development of coating, cosmeceutical, and biomedical applications.

## 1 Introduction

Resource efficiency, meaning doing more and better with less, is an important aspect of material development and a key challenge for circular processes. The diversity found in bioresources gives rise to considerable variation in composition, biofunctions, and robustness of their cell wall structure (Gibson, 2012). This, in turn, will dictate how much effort is required to isolate building blocks from bioresources and pose challenges in maximizing recovery when refining and utilizing these resources (Huppes and Ishikawa, 2005) and developing them into functional high-value-added materials (Zhang et al., 2025).

Cacao prices reached historical and record high values, $10.75 per kilogram, and the lowest production levels in 46 years in 2025 (The International Cocoa Organization, 2025). The husk of the cacao bean (*Theobroma cacao* L.), also referred to as bean shell, is the main by-product generated during chocolate production. For every kilogram of chocolate produced, more than 100 g of cacao bean husk is obtained (The International Cocoa Organization, 2025). Today it is estimated that 700,000–900,000 tons of cacao bean husk are generated worldwide, approximately 300,000 of which are found in Europe (Sánchez et al., 2023). The critical issue in cacao production is the handling of husk residues, which are often discarded or burned, contributing to environmental challenges.

Cacao processing residue is an underutilized industrial by-product that can be converted into value-added materials, thereby supporting more resource-efficient, circular use of industrial side streams. Research on valorization of cacao residue includes extraction of pectin (Muñoz-Almagro et al., 2019) and nanocellulose (Souza et al., 2019), fat-rich emulsions (Gómez Hoyos et al., 2023), additives in food processing as an adsorbent or corrosion-inhibiting agent (Sánchez et al., 2023), and phenolic-based antioxidants (Dewi et al., 2025). Some efforts have used cacao residues in low-value applications, including husk tea, animal feed, mulch, and fertilizer (Gutiérrez-Macías et al., 2021). There is a growing exigency to use cacao residues for value-added applications from an environmental and economic perspective, including a societal and cacao/chocolate producer need.

Today, cellulose nanofibers are mainly produced from wood pulp with additional chemical pretreatments performed to purify cellulose prior to fibrillation (Ai et al., 2022). In general, research and industrial processes for reaching nanoscale are associated with several processing steps that include the use of chemicals, high energy consumption, and low production efficiencies being the largest contributors to the environmental impact (Berglund et al., 2020; Ai et al., 2022). On the other hand, we have in previous studies reported that cellulose nanofibers from different forest-based and industrial bioresidues have been separated in the presence of non-cellulosic components such as lignin, hemicellulose, pectin, and alginate, with increased yield and efficiency (Hassan et al., 2018; Berglund et al., 2020; Berglund and Sharma, 2026). Making use of the natural composition that the biomass offers, in addition to reducing fractionation or pretreatment steps, has the potential to promote energy-efficient conversion and contribute to an overall lower environmental impact for carbon footprint, freshwater ecotoxicity, and human toxicity, along with a cost reduction when developing nanofibers from bioresidues (Berglund et al., 2020).

Although many research studies have reported on the use of various bioresources and residues and their fibrillation into cellulose nanofibers, only a few previous studies have examined the development of nanofibers from cacao residue. For example, Souza et al. (2019) reported on the direct fibrillation of cacao residue into fibers and its rheological properties, where the fibrillated material exhibited a weak gel characteristic with brown coloration (Souza et al., 2019). Gómez Hoyos et al. (2020) fibrillated nanofibers from cacao residue with several extensive pretreatment steps, including potassium hydroxide (KOH), followed by sodium chlorite (NaClO_2_) and acetic acid (CH_3_COOH), followed by a second KOH treatment and finally hydrochloric acid (HCl), and with a yield of only 7%.

When considering cacao residue as a bioresource, there is a very rich natural composition to make use of, including cellulose, hemicellulose, lignin, pectin, and phenolic compounds, and significant fat content with a lipid profile very similar to that of cocoa butter (Okiyama et al., 2017). It is essential to understand how the natural composition and bioactive compounds affect the processing of cacao residue into nanofibers. Exploring the functionality is key to developing resource-efficient and functional materials.

The hypothesis of this work is that the intrinsic composition of cacao residue can be utilized to a larger extent for increased resource efficiency upon fibrillation into nanofibers and to separate nanofibers with specific functionality. The aim was to study the processing and properties of cacao nanofibers for a better understanding of their structure–process–property relationship. The process was evaluated according to yield and energy consumption, and the chemical composition was studied before and after pretreatment. Cacao nanofiber characteristics such as dimensions, network formation, redispersibility, and wettability were evaluated using microscopy, rheology, mechanical properties, and contact angle measurements. The potential to achieve bioactive cacao nanofibers in the process included antioxidant, antimicrobial, and anti-collagenase activity evaluation, and lysozyme degradation. To evaluate the potential of cacao nanofibers, fibrillation of nanofibers from commercial wood pulp was carried out and used as a reference material in this study.

## 2 Materials and methods

### 2.1 Materials

Cacao residue, a by-product after chocolate production, was kindly provided by *Nordic Chocolate AB*, *Umeå*, *Sweden*, and used as the raw material in this study*.* Sodium chlorite (NaClO_2_), sodium hydroxide (NaOH), and acetic acid (96% CH_3_COOH) were purchased from Merck Life Science AB, Solna, Sweden. All chemicals were used as received. Deionized water was used for all experiments. A commercial birch kraft pulp was used as a reference material, kindly supplied by *SCA Munksund*, *Piteå*, *Sweden*.

### 2.2 Preparation of cacao nanofibers

The pretreatment target was to reduce the lignin, while preserving other natural components of the cacao residue. The residue was soaked (50 g cacao per 500 mL water) 24 h prior to pretreatment by bleaching with NaClO_2_ (1.7%) in an acetate buffer (pH 4.5) at 80 °C for 2 h. The pretreatment was performed twice, followed by washing until a neutral pH was reached. The solid recovery was calculated as a yield according to Equation 1:

$$Yield\left(\%\right)=\left(\frac{W_{1}}{W_{0}}\right)\times 100,$$ (1)

where W_1_ indicates the dry weight of the sample after the purification and W_0_ denotes the initial dry weight of the cacao residue. The presented yields are based on the average of ten different batches.

The materials (at a concentration of 3 wt.%) were fibrillated using an MKZA6-3 ultrafine friction grinder (Masuko Sangyo® Co., Ltd., Kawaguchi, Japan) with coarse silica carbide (SiC) grinding stones. The fibrillation was conducted in contact mode, with the gap between the two discs gradually set to −90 μm, at 1,500 rpm.

The energy demand of the fibrillation process was calculated from the product of the power, measured with an energy analyzer, EM24 DIN (Carlo Gavazzi, Belluno, Italy), and the processing time, and expressed as kilowatt-hour per kilogram of dry weight of nanofiber. Samples were collected at regular intervals to assess the degree of fibrillation using viscosity as an indirect measurement of separated fibers that display a stronger gel formation and optical microscopy to detect larger structures. The process was finalized when a viscosity plateau was reached, and/or no larger intact structures could be observed in optical microscopy analysis.

### 2.3 Chemical composition

The chemical composition of raw cacao residue and pretreated cacao residue was determined to study the effect of pretreatment and to connect the composition to the characteristics of cacao nanofibers. The cacao residue was ground to a fine powder with an IKA A11 basic analytical mill (IKA, Staufen, Germany), and the pretreated cacao residue was freeze-dried prior to chemical composition analysis from the mass fraction of extractives, structural carbohydrates, and total lignin following the method previously described (Berglund et al., 2024). The mass fraction of the samples was based on the average values of triplicate readings to calculate the dry matter content of the respective samples. The ash content was determined by calcining 200 mg dry samples by heating to 575 °C with a ramping temperature of 5 °C per min and held for 3 h. The difference between the initial and final weights of the crucibles was used to determine the ash content in the samples.

### 2.4 Optical microscopy

An optical microscope, Nikon Eclipse LV100N POL (BergmanLabora AB, Danderyd, Sweden), and the imaging software NIS-Elements D 4.30 were used to study how the fibrillation process affected the material structure and its micrometer-scale size.

### 2.5 Rheology

Viscosity measurements were performed during the fibrillation process to evaluate the degree of fibrillation using a Vibro Viscometer SV-10 (A&D Company, Ltd., Oxford, United Kingdom) at a constant shear rate and with periodical circulation of the sensor plates from zero to peak (sine-wave vibration) at a frequency of 30 Hz. The temperature increased during the fibrillation owing to the compression and abrasive shearing forces; hence, the viscosity measurements were repeated at a temperature of 22.3 °C ± 1.0 °C to confirm that a viscosity plateau had been reached during the process. The presented values are an average of three measurements for each sample. The fibrillated gels were further characterized at a solid content of 2 wt.% at 22.5 °C ± 0.5 °C using a Discovery HR20 rotational rheometer (Waters AB, Solna, Sverige) with a 25 mm parallel plate geometry. A gap of 1 mm was maintained for all tests. A shear rate range between 0.01/s and 1,000/s was used for flow sweep analysis. An oscillatory frequency sweep was conducted at a constant strain of 1%, varying the frequency from 0.01 Hz to 10 Hz. A texture analysis using a CT3 texture analyzer (Cromocol Scandinavia AB, Borås, Sweden) was performed to further analyze cacao and reference nanofibers using the normal use test mode, with a trigger force of 0.009 N, a deformation distance of 15 mm, and a test speed of 1 mm/s, employing a cylindrical probe (TA10) with a diameter of 12.7 mm and a length of 35 mm, at room temperature (approximately 23 °C). The materials were characterized at 2 wt.% concentration.

### 2.6 Scanning electron microscopy

The microstructure of the raw cacao residue and the pretreated and fibrillated material was examined using a JEOL JSM-6460LV scanning electron microscope (JEOL Nordic AB, Sollentuna, Sweden) at an acceleration voltage of 15 kV. All samples were coated using a Leica EM ACE200 sputter-coater machine (Wetzlar, Germany) with a gold target to prevent electron charging. The coating was performed in a vacuum of approximately 6 × 10^−5^ mbar under a current of 100 mA for 20 s to obtain a coating thickness of 10 nm.

### 2.7 Atomic force microscopy (AFM)

The cacao nanofibers were studied using AFM. The fibrillated sample suspension (0.01 wt.%) was dispersed and deposited by spin coating onto a clean mica surface for imaging with a Veeco Multimode Scanning Probe (Santa Barbara, United States) in tapping mode, with a Bruker tapping mode etched silicon probe with aluminum (TESPA) tip (antimony-(n-)doped silicon) (Camarillo, United States). The nanofiber size (width) was measured from the height images using the Nanoscope V software.

### 2.8 Size exclusion chromatography (SEC)

Pretreated cacao residue and cacao nanofibers were analyzed according to the molecular weight distributions (MWDs) of their carbohydrate polymers using size exclusion chromatography (SEC). The samples were freeze-dried and directly dissolved in DMAc (8% LiCl) and diluted five times (to 1.6% LiCl) and filtered through 0.45 PTFE filters before analysis. The MWDs of the carbohydrate polymers were analyzed in DMAc with 1% LiCl as the solvent and mobile phase. The SEC system consisted of a guard column and two serial T6000M columns from Malvern. The temperatures were set at 50 °C, and the flow rate was set to 0.5 mL/min. The residence times were detected using a refractive index detector, and the columns were calibrated using seven Pullulan standards dissolved in the mobile phase. Polystyrene standards dissolved in the same solvent were used to calibrate the detectors. The data were evaluated using the OMNISEC V11 software from Malvern.

### 2.9 Antioxidant activity

The 2,2-diphenyl-1-picrylhydrazyl (DPPH) radical scavenging activity of the cacao and reference nanofibers was assayed according to Xu et al. (2021). Freeze-dried samples were weighed in a tube, and 0.4 mL of water was added and shaken vigorously. Then, 0.4 mL of 0.2 mM DPPH ethanol solution was added. The final concentration of freeze-dried sample varied between 0.0625 mg/mL and 12.5 mg/mL. The final concentration of DPPH was 0.1 mM. A blank sample was prepared containing 0.4 mL water and 0.4 mL of 0.2 mM DPPH ethanol solution. A series of blank samples was prepared containing nanofibers without DPPH and adding 0.4 mL water and 0.4 mL ethanol. The final mixtures were allowed to shake at 1,000 rpm and room temperature in the dark. At 30 min and 20 h, an aliquot was withdrawn, and the sample mixture was centrifuged for 2 min at 5,000 rpm to remove the sample solids. The supernatant was pipetted into a 96-well plate, and its absorbance at 517 nm was measured via a microplate reader. The DPPH radical scavenging activity was calculated following Equation 2:

$$DPPH\;rapicals\;scavenging\;activity\;\left(\%\right)=\left(1-\frac{\left(A_{s}-A_{1}\right)}{A_{0}}\right)\times 100,$$ (2)

where A_s_ is the absorbance of the sample with DPPH, A_1_ is the absorbance of the sample (nanofiber in water:ethanol 50:50 v/v), and A_0_ is the absorbance of the control (DPPH 0.1 mM in water: ethanol 50:50 v/v). The concentration of antioxidants required to reduce the initial concentration of DPPH free radicals by 50% is defined as the IC_50_ value.

### 2.10 Leaching of bioactive compounds

Cacao and reference nanofibers were freeze-dried, and 20 mg of the samples were incubated in 20 mL of phosphate buffer (0.1 M, pH 5.8 or pH 7.4) over a period of 14 days. A homogeneous sample was withdrawn at daily intervals and centrifuged. The supernatant was collected for assessment of total phenolics using the Folin–Ciocalteu method, and antioxidant activity was determined using the DPPH assay.

### 2.11 Degradation by lysozyme and collagenase inhibition

For further studies on bioactivity, 15 mg of freeze-dried cacao and reference nanofibers were added to tubes containing 900 μL of phosphate buffer (0.1 M, pH 7.4). A 100 μL aliquot of stock human lysozyme solution (Merck, Germany) was added so that the final enzyme concentration was 1 mg/mL reaction volume. The reaction was incubated at 37 °C and 1,200 rpm for 14 days. The reaction was terminated by centrifugation (10 g for 10 min), removing the supernatant and washing the remaining material thoroughly with Milli-Q water for at least three cycles. The sample was subsequently placed in an oven at 50 °C for 48 h. Reactions prepared with sample, buffer, and water instead of the enzyme were carried out as negative controls.

The degradation (%) was calculated following Equation 3:

$$Degradation\;\left(\%\right)=\frac{\left(m_{tf}{-m}_{t0}\right)\text{lysozyme}\times 100}{\left(m_{tf}{-m}_{t0}\right)\text{negative}\;\text{control}}.$$ (3)

The inhibition of collagenase was assessed by a colorimetric kit (Abcam, United Kingdom). In short, freeze-dried material was placed in Eppendorf tubes (0.25–5 mg) containing 400 μL assay buffer, and 40 μL of collagenase stock solution was added. The samples were incubated at room temperature and 800 rpm for 20 min, followed by centrifugation (10 g for 10 min), and the supernatant, containing the enzyme, was collected. The residual activity of the enzyme was measured. The supernatant (100 μL) was added to 110 μL of the substrate mixture, and the absorbance was measured at 345 nm and 37 °C for 20 min in a SpectraMax reader (Molecular Devices, United States). The activity assay was also carried out on non-inhibited enzyme as the positive control, and on a sample containing only nanofibers without enzyme to exclude any background interference of the material during the assay measurement. The % inhibition was defined as shown in Equation 4:

$$\%Inhibition=\frac{\left[\left({Initial\;rate}_{non-inhibited}\right)-\left({Initial\;rate}_{inhibited}\right)\right]\times 100}{\left({Initial\;rate}_{non-inhibited}\right)},$$ (4)

where the initial rate is calculated as the slope of the linear decrease in absorbance over time: ΔΑ/Δt.

### 2.12 Antibacterial activity

The antimicrobial activity of the cacao and reference nanofibers was tested against *Escherichia coli* BL21. A preculture was prepared in sterilized Luria–Bertani (LB) medium and incubated for 4 h at 37 °C until the optical density at 600 nm reached 0.600. Then, 100 mL sterilized flasks containing 20 mL of medium were inoculated with 1% (v/v) of the active preculture and incubated at 37 °C for up to 24 h. The culture medium consisted of LB medium containing nanofiber gel at concentrations of 1%, 5%, or 25% (v/v). Cultures containing LB and water instead of the sample were prepared as positive controls. After inoculation, samples were withdrawn under sterilized conditions at 0, 4 h, and 24 h and diluted 10 times. Their optical density was measured at 600 nm. The bacterial growth was quantified as follows Equation 5:

$$Relative\;bacterial\;growth\;\left(\%\right)=\frac{\left({OD600}_{tf}-{OD600}_{t0}\right)nanofiber\times 100}{\left({OD600}_{tf}-{OD600}_{t0}\right)positive\;control}.$$ (5)

### 2.13 Redispersion

Networks were prepared from cacao and reference nanofiber suspensions at 250 g/m^2^ using vacuum-assisted filtration and were dried according to a previously reported procedure (Berglund et al., 2016). Following the study carried out by Siqueira et al. (2016), redispersion was carried out with a suspension of 1.5 wt.%. The dried networks were cut into small pieces using scissors. The suspension was prepared with distilled water and mixed using an Ultra-Turrax at 10,000 rpm for 5 min. Viscosity measurements and optical microscopy were used to evaluate the cacao nanofibers and reference, before and after redispersion. Viscosity before and after redispersion was compared using Welch’s independent-samples t-test using PAST version 4.17 (Natural History Museum, University of Oslo, Oslo, Norway). The normality of the data was assessed using the Shapiro–Wilk test, and statistical significance was accepted at p < 0.05. Data are presented as mean ± standard deviation (n = 3).

### 2.14 Mechanical testing

The effect of redispersion on the network formation ability was further evaluated by mechanical testing in tensile mode for the initially prepared networks and the ones prepared after redispersion. The sample preparation and the tensile testing procedure were previously reported (Berglund et al., 2016). An AG X tensile testing system (JK Lab Nordic AB, Åkersberga, Sweden) equipped with a 1 kN load cell was used at an extension rate of 2 mm/min and with a gauge length of 20 mm. All samples were conditioned at 50% ± 2.0% relative humidity at 22.1 °C ± 1.0 °C for 48 h. Statistical analyses were performed using PAST version 4.17 (Natural History Museum, University of Oslo, Oslo, Norway). The normality of the data was assessed using the Shapiro–Wilk test. Because all datasets were normally distributed (p > 0.05), differences in tensile strength, strain at break, and elastic modulus between the two materials were evaluated using Welch’s independent-samples t-test. Statistical significance was set at p < 0.05, and data are presented as mean ± standard deviation, n = 10 per material.

### 2.15 Water contact angle

The wettability of the nanofiber networks was characterized using water contact angle measurements with an EASYDROP measuring system, drop shape analysis control (DSA1), and evaluation software (Krüss GmbH, Hamburg, Germany). A 4 μL water drop was placed onto the samples, and the contact angle was calculated using the sessile drop technique. Water contact angle data (n = 10 per material) were analyzed using Welch’s independent-samples t-test in PAST version 4.17 (Natural History Museum, University of Oslo, Oslo, Norway) after confirming normality with the Shapiro–Wilk test. Statistical significance was set at p < 0.05, and data are presented as mean ± standard deviation.

## 3 Results and discussion

### 3.1 From cacao residue to cacao nanofibers

For a better understanding of the structure–process–property relationship from cacao residue to cacao nanofibers, the chemical composition was characterized before and after pretreatment, that is, bleaching the cacao residue with NaClO_2_. The results from chemical composition are presented in Table 1, and detailed hemicellulose and fatty acid composition is provided in Supplementary Tables S1-S2. The idea was to pretreat cacao residue to reduce lignin while preserving other natural components, and to study the effect of preserving the non-cellulosic components of cacao residue on the fibrillation process and the properties of cacao nanofibers.

**TABLE 1:** Chemical composition of cacao residue before and after pretreatment in (w/w), with standard deviations.

Component	Cacao residue (w/w)	After pretreatment (w/w)
Glucan (cellulose)	9.6 ± 0.3	26.0 ± 0.2
Hemicellulose	3.6 ± 0.1	6.8 ± 0.0
Pectin^*^	3.1 ± 0.2	3.0 ± 0.1
Hexane extracts (fat)	29.9 ± 0.4	41.8 ± 0.8
Ethanol extracts	3.2 ± 0.	8.1 ± 0.1
Acid-insoluble lignin (Klason lignin)	15.9 ± 1.1	1.2 ± 0.2
Acid-soluble material	14.1 ± 0.7	3.8 ± 0.8
Ash	4.0 ± 0.1	-

* Pectin material consists of rhamnose and galacturonic acid.

Cacao residue was found to be 16% carbohydrates, including glucan (cellulose), hemicellulose, and pectin, as shown in Table 1. The glucose released during analytical acid hydrolysis could be attributed to cellulose; however, the analysis does not distinguish between cellulose, starch, and other possible glucans. On the other hand, the starch content of cacao residue is expected to be present in minor (Rojo-Poveda et al., 2009) to negligible amounts (Vriesmann et al., 2011) for bean husk; thus, the major component is presumed to be cellulose. Sánchez et al. (2025) reported that cellulose (measured as glucan content) was the major sugar detected at an average of 14% of total cacao residue biomass (Sánchez et al., 2025).

As detailed in Table 1, cacao residue extractives accounted for approximately 34%, of which non-polar extractives consisted of fat (30%), being the major chemical component of cacao residue, which had stearic, oleic, palmitic, and oleic acids as the major fatty acids (esterified fatty acids, Supplementary Table S2). Regarding the lignin (Klason lignin and acid-soluble material) (30%) and ash (4%), the obtained values are in accordance with those reported by Sánchez et al. (2025). However, the sum of all identified components does not reach 100%. The lack of closure might be attributed to the fact that the analytical procedure did not include the quantification of either soluble sugars or protein, which together can represent up to 19% (w/w) of cacao residue dry mass (Vriesmann et al., 2011), or acetyl groups, which are hemicellulosic constituents whose relevance varies depending on the biomass type.

After pretreatment, the lignin fractions were considerably reduced to approximately 5%, and hence the samples primarily consisted of carbohydrates and extractives, as provided in Table 1. The carbohydrate content after pretreatment (36%) was more than double that of the content in raw cacao residue. The total extractives content of pretreated cacao residue was approximately 50%, which is considerably higher than before bleaching. The bleaching also led to a significant reduction in the share of stearic acid and to a relative increase in the share of the other acids (Supplementary Table S2). It has previously been reported that the cell wall cleavage promotes the liberation of other components, such as triacylglycerides (three fatty acids chemically linked to a glycerol molecule) and that the modification or pretreatment of cell wall polymers (e.g., pectic polysaccharides and hemicelluloses) increases the extraction of oil or fat in, for example, almonds (Femenia et al., 2001) and grape seeds (Passos et al., 2009). It should also be noted that the chemical composition of cacao residue, including fat and bioactive compounds, varies depending on cacao source (Sari et al., 2024).

The yield after pretreatment of cacao residue was 30 wt.% ± 11, measured from 10 separately pretreated batches. Gómez Hoyos et al. (2020) reported a yield of 7 wt.% after multiple pretreatment steps including: KOH solution, NaClO (bleaching), KOH solution, and finally HCl solution for the preparation of suspensions of holocellulose nanofibers and fat. The same study reported the presence of functional groups from cellulose, hemicellulose, and fat using ATR-FTIR and NMR characterization. The results from chemical composition analysis in this study emphasize that the intrinsic components of cacao residue were successfully preserved after pretreatment, simultaneously as the lignin content was reduced, thus enabling further study on the effect of non-cellulosic components on the fibrillation into cacao nanofibers and their characteristics.

Microscopy analysis of the material structure was carried out to study the effect of pretreatment and fibrillation of cacao residue compared to a reference material: birch kraft pulp. Figure 1 shows photographs of the prepared materials from the cacao residue and the reference material, including the gel obtained after fibrillation and the dried networks prepared for the redispersion study. The fibrillation process of the materials was further evaluated according to energy consumption and network formation ability. Viscosity measurements as a function of the energy demand and OM images captured during the fibrillation process are shown together with viscosity as a function of shear rate and stress in Figure 1, and OM images at different magnifications are provided in Supplementary Figures S1-S2.

**FIGURE 1:**
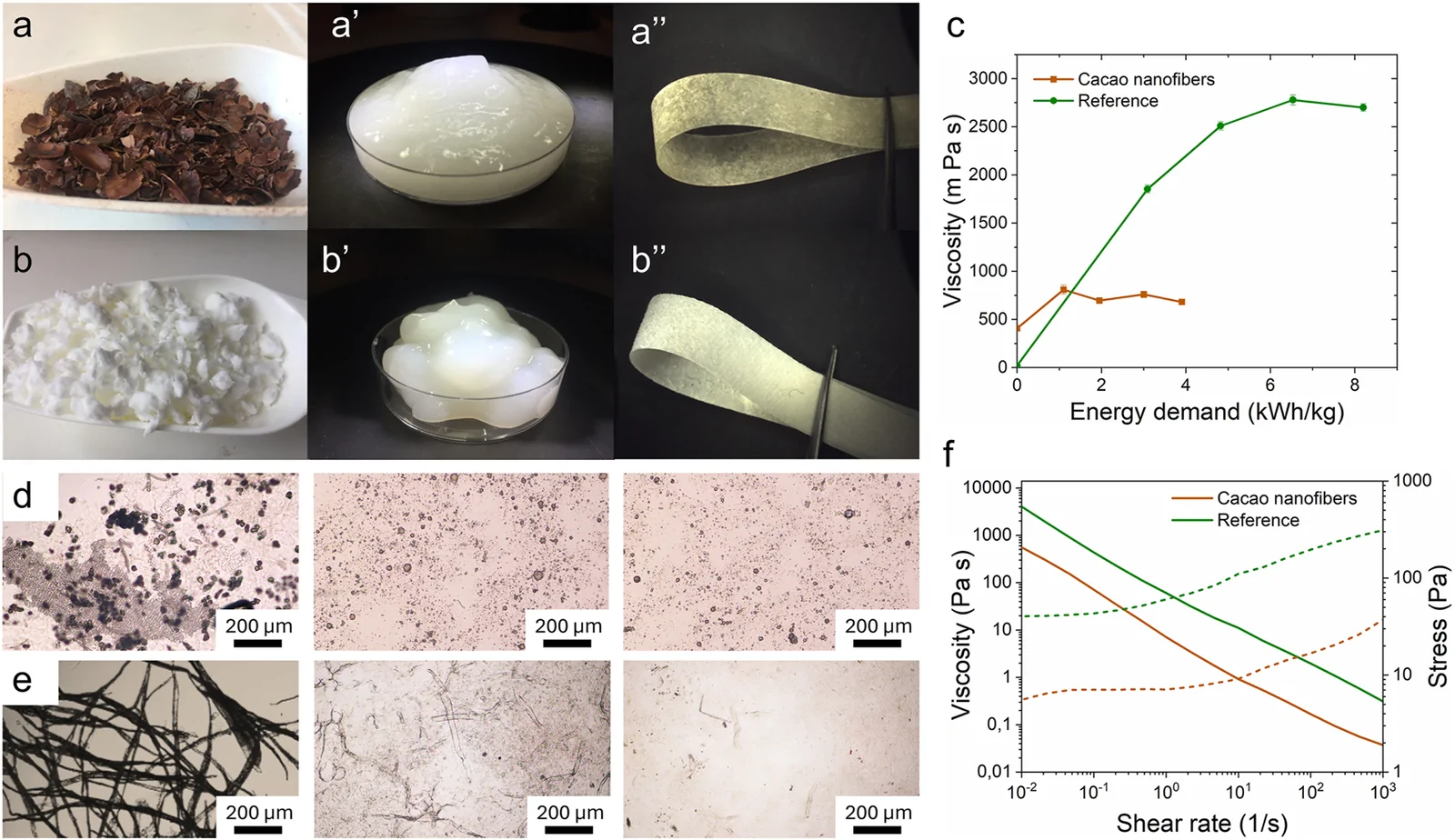
Photographs of the materials **(a)** cacao residue, **(a’)** gel of cacao residue after pretreatment and fibrillation, and **(a’’)** dried nanofiber network. **(b)** Reference material: birch kraft pulp, **(b’)** gel of reference material after fibrillation, and **(b’’)** dried nanofiber network. **(c)** Viscosity as a function of the energy demand during fibrillation, and OM images of samples collected during fibrillation of **(d)** cacao residue and **(e)** reference material. **(f)** Viscosity (continuous line) as a function of shear rate and stress (non-continuous line)

From Figure 1, both starting materials (a, b) formed gels after fibrillation (a’, b’) and could successfully be formed into dried networks (a’’, b’’). Measurements of viscosity and energy consumption were performed at regular intervals during fibrillation (Figure 1c) with the intent to indicate the degree of separation into fibrils. In general, the fibrillation process results in a continuous increase in the viscosity and is carried out until a plateau in the viscosity is observed, connected to the disintegration of fibrils forming a stronger network (Berglund et al., 2016).

The fibrillation processes were performed at comparable concentrations, settings, time, and batch sizes. A clear difference in the fibrillation process was observed between the materials, where pretreated cacao residue displayed a viscosity of 682 ± 24 mPa⋅s at an energy demand of 3.9 kWh/kg. The reference material was found to have a viscosity of 2,693 ± 38 mPa⋅s, corresponding to an energy consumption of 8.2 kWh/kg after the fibrillation process was finalized (Figure 1c). Fibrillation of cacao residue showed an initial viscosity increase upon fibrillation, with the highest viscosity at 810 ± 39 mPa⋅s; however, the OM images show that at this point there were still larger intact structures present (Figure 1d). Upon further fibrillation, the reduction of larger intact structures was observed from OM images, in which the fat is apparent as smaller black spots and clusters. For cacao residue, fibrillation did not result in a continuous increase of viscosity, as shown in Figure 1c, indicating that the fat particles hinder connection points between fibrils in the gel that allow for strong network formation. The reference material displayed typical behavior during fibrillation and increased continuously to a plateau, and the OM images displayed a size reduction and absence of larger intact structures after fibrillation. It is plausible that the preserved high fat content in pretreated cacao residue also affects the fibrillation process into smaller structures, where we hypothesize that the retained fat may act to decrease the friction between the grinding stones, which apply shearing forces through the contact between one stationary and one rotating stone. If so, this mechanism could contribute to the more than 50% lower energy demand measured for the cacao residue compared to the reference material.

To study the potential for cosmetic applications, further characterization of rheological behavior was carried out and showed that both gels exhibit shear-thinning behavior, meaning their viscosity decreases as the shear rate increases (Figure 1f), and confirmed the lower viscosity of cacao nanofibers compared to the reference nanofibers, as also observed at constant shear rate (Supplementary Figure S4a). Oscillatory frequency sweep provided in Supplementary Figure S4b showed that both materials formed a solid gel network (Brummer, 2006), as indicated by the storage modulus (continuous line) being significantly higher than the loss modulus (non-continuous line) across the entire frequency range. However, cacao nanofibers exhibit lower moduli and less stress to initiate flow than the reference, in agreement with the effect of fat reducing internal friction. Thus, cacao nanofibers as a viscosity-reducing agent in lotions or creams could facilitate pumping or dispensing from cosmetic containers (Brummer, 2006). In addition, texture analysis was performed to predict consumer sensory perception (Supplementary Figure S5; Supplementary Table S3). The cacao nanofibers displayed lower work values and adhesiveness, indicating better spreadability, for example on the skin, and a less “sticky” material compared to reference nanofibers.

To further investigate the cacao structure during processing, the raw cacao residue, before and after pretreatment and fibrillation, was characterized using SEM. The fibrillated material was also studied with AFM for further understanding of the material at a nanoscale. The microscopy analysis of cacao residue during the various stages of processing is shown in Figure 2.

**FIGURE 2:**
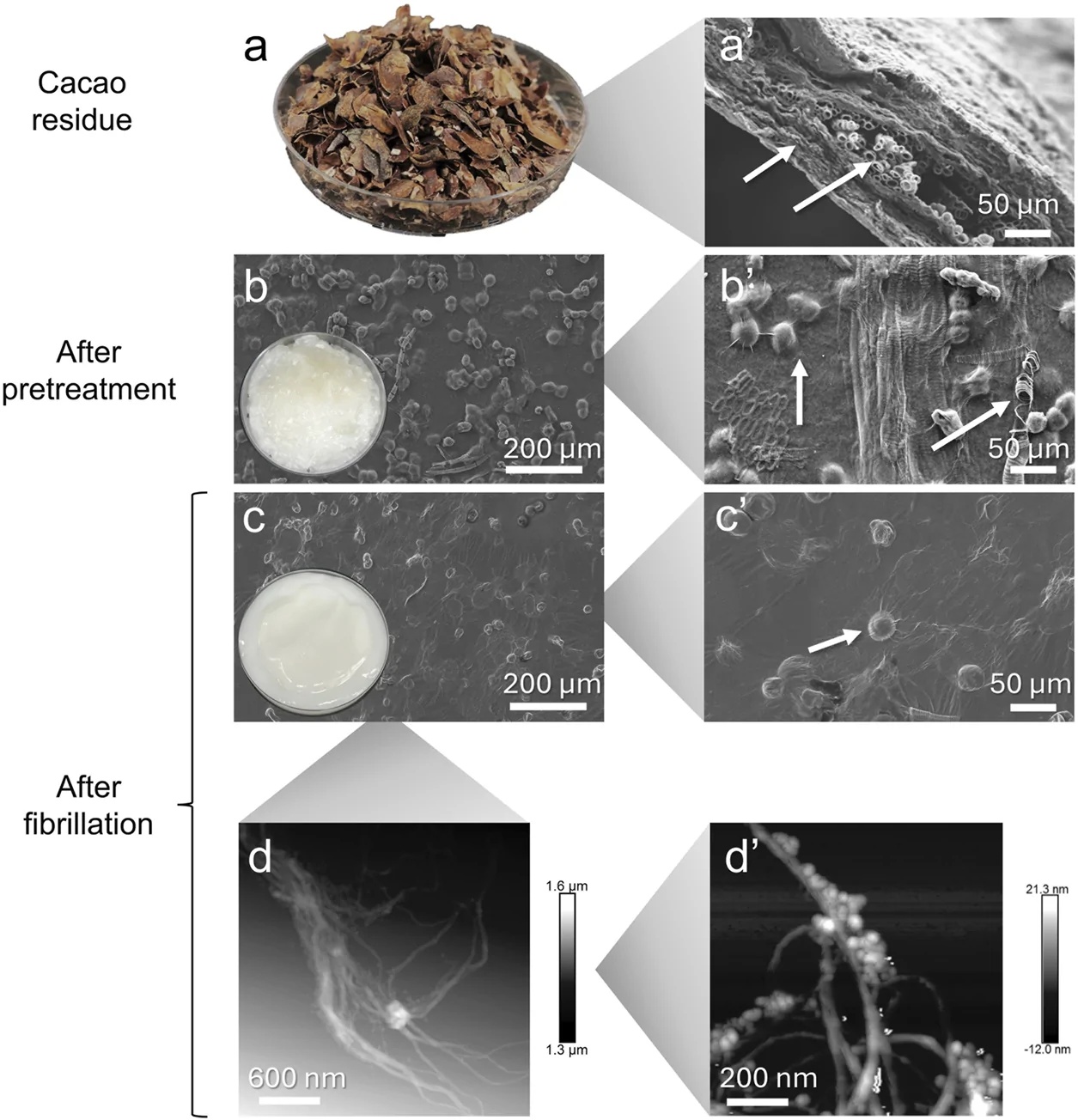
**(a)** Photograph and **(a’)** SEM image (cross-section), magnification ×100, of the cacao residue used as starting material. SEM images of pretreated cacao residue **(b, b’)** before fibrillation, and **(c, c’)** after fibrillation, at ×300 and ×100 magnification, respectively (corresponding photographs, inset). **(d, d’)** AFM height images after fibrillation.

The cacao residue (Figure 2a) was analyzed using SEM. The cross-section displayed a layered structure (sclerenchyma cells), with tracheid elements, visibly detected in the middle of the layers as circular hollow structures (indicated with arrows, Figure 2a’), that have previously been described for cacao residue (Gómez Hoyos et al., 2020). After pretreatment, the presence of spiral-formed cells was observed from SEM (Figures 2b,b’) and OM images (Supplementary Figure S3). These cells have been shown in a previous study of cacao residue after pretreatment with acid maceration (Souza et al., 2019). Notable after pretreatment was the cacao fat that appears in abundance as large spherical particles or globules, as shown in Figures 2b,b’. After fibrillation, the spherical particles are still visible, although less pronounced, and the absence of larger intact structures was observed (Figures 2c,c’).

AFM analysis revealed the presence of nanofiber structures (measured in the range of 5–20 nm) after fibrillation, as observed from height scans in Figures 2d,d’. AFM showed smaller spherical particles appearing in clusters (Figure 2d) and covering the nanofiber surfaces (Figure 2d’), which indicate that the size of the fat particles also was partly reduced upon fibrillation. Measurements with AFM are not a representative analysis for overall fiber dimensions because larger structures are overlooked, but they can provide information on a nanoscale. Previous studies on fibrillation from cocoa bean shells have presented nanoscale dimensions smaller than 100 nm. For example, Souza et al. (2019) reported nanofiber dimensions between 5 nm and 60 nm after a combination of homogenization and mechanical fibrillation using a Masuko grinder (Souza et al., 2019). Gómez Hoyos et al. (2020) described nanofiber dimensions in the range of 30–80 nm after multiple chemical pretreatments followed by mechanical fibrillation using a Masuko grinder. The same study also showed the presence of smaller spherical fat particles after fibrillation (Gómez Hoyos et al., 2020).

The effect of fibrillation was further studied with SEC and numerical evaluation of the molecular weight distribution (Supplementary Table S3; Supplementary Figure S6) of pretreated cacao residue and cacao nanofibers. Two distinct peaks were observed. The lower peak is dominated by hemicelluloses (Galland et al., 2015). The cacao nanofibers showed a narrower molecular weight distribution after fibrillation compared to the pretreated cacao residue, as indicated by lower polydispersity indices (PDI) of 11 and 18, respectively (Supplementary Figure S6; Supplementary Table S4). Iwamoto et al. (2007) showed that repeated passing of dissolving pulp fibers through a Masuko grinder resulted in reduced degree of polymerization, connected with rupturing of long fibrils. In the present study, the decreased M_w_ of the cellulose is consistent with increasing degradation with the fibrillation processing in the Masuko grinder, while cacao nanofibers appear to have a hemicellulose fraction of intact M_w_ (position of low M_w_ peak) compared to that of pretreated cacao residue.

### 3.2 Cacao nanofiber characteristics

#### 3.2.1 Redispersion and network formation

To study the redispersion ability of the cacao and reference nanofibers, their network formation was assessed before and after redispersion using OM and viscosity, as were the mechanical properties of the initially prepared dried networks and the network prepared after redispersion (Supplementary Figure S7). The results are presented in Figure 3.

**FIGURE 3:**
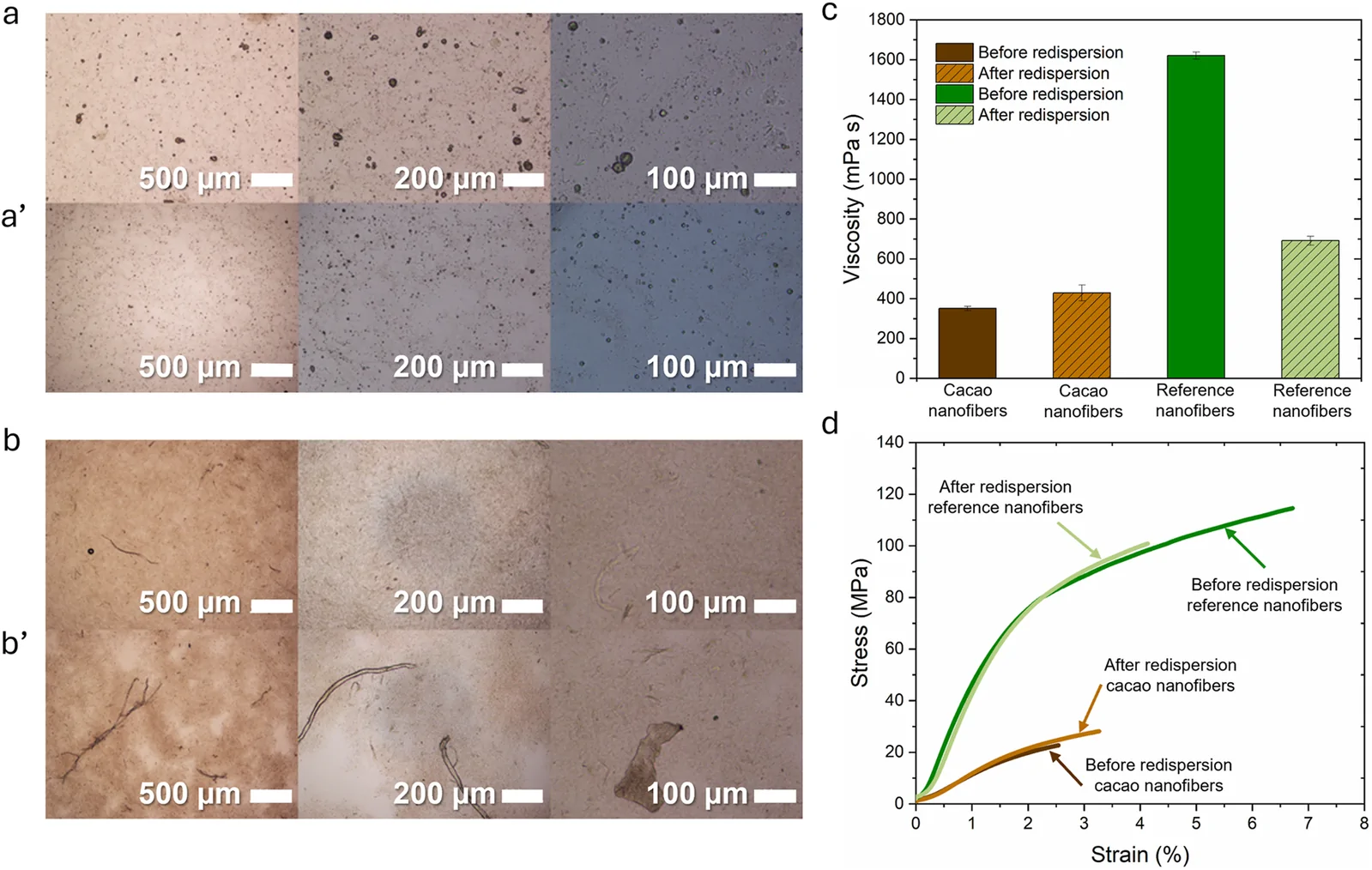
OM images at 1.5 wt.% of **(a)** cacao nanofibers, **(b)** reference nanofibers, and after drying and redispersion **(a’)** cacao nanofibers and **(b’)** reference nanofibers. **(c)** Viscosity measurements at 1.5 wt.% before and after dispersion. **(d)** Representative stress and strain curves of dried networks tested in tensile mode for cacao and reference nanofibers, respectively.

Figures 3a,b show the OM images before drying and redispersion of the cacao nanofibers and reference nanofibers, respectively. After drying and redispersion, the cacao nanofibers (Figure 3a’) did not show any visible larger intact structures, nor a tendency for agglomerates, compared to the micro-sized fiber structures observed for the reference nanofibers after redispersion (Figure 3b’). From Figure 3c, the cacao nanofibers showed overall lower viscosity than the reference material. The hydroxyl group-rich surfaces of cellulose nanofibers have a tendency toward high water adsorption and swelling, and thus strong gel formation (Solhi et al., 2023), which was apparent for the reference material, displaying high viscosity, where no fat is affecting the network formation.

Redispersion affected the viscosity of the two materials differently (Figure 3c). The viscosity of the cacao nanofiber suspension displayed an increased trend from 351 ± 5 to 427 ± 36; however, no statistically significant change (Supplementary Table S5). In contrast, the viscosity of the reference material decreased significantly from 1,610 ± 24 to 696 ± 37 after redispersion (Supplementary Table S5), indicating that the drying of reference nanofibers results in loss of their nanoscopic dimensions, leading to deterioration of their network-formation ability. Similar redispersion behavior was also observed for the mechanical properties of the dried networks, presented in Figure 3d. For the cacao nanofiber network, tensile strength and strain at break increased significantly following redispersion, whereas the elastic modulus remained unchanged (Supplementary Table S6). In contrast, the reference network exhibited significant reductions in tensile strength and strain at break after redispersion, while the elastic modulus was not significantly affected (Supplementary Table S6).

In a previous study, never-dried and dried paper mill sludges were compared for nanofiber production and showed that paper mill sludge could be dried before fibrillation with comparable properties to reduce transportation and storage challenges posed on an industrial scale (Adu et al., 2018). However, for cellulose nanofibers, which are dried and redispersed, their nanoscale dimensions are generally lost due to pronounced association, caused mainly by attractive van der Waals forces and the well-known hornification of cellulose-rich fibrous materials (Eyholzer et al., 2010).

The mechanisms leading to aggregation have been previously studied, and strategies to inhibit or prevent hornification and improve redispersibility have been reported. For example, Iwamoto et al. (2008) showed that the presence of hemicellulose for pulp-derived nanofiber preparation was beneficial to prevent hornification (Iwamoto et al., 2008). Siqueira et al. (2016) reported on redispersible carrot nanofibers where the surface chemistry, that is, the presence of hemicellulose and pectin, played a key role in redispersibility. Visanko et al. (2017) reported on the fabrication of redispersible high-lignin-containing nanofibers. By increasing the surface charge density of cellulose nanofibers or adding lignin as a redispersing agent, Nordenström et al. (2021) showed comparable mechanical properties measured for the dried network before and after redispersion. In this study, the purer composition of the reference nanofibers, with cellulose as the main component, can be expected to result in abundant contact points and enhanced network strength through extensive hydrogen bonding between the nanofibers. In contrast, the presence of non-cellulosic components in the cacao nanofibers, particularly the retained fat fraction, may impair the formation of contact points and reduce the strength of inter-fibrillar bonding. At the same time, the retained fat may plausibly contribute to preventing hornification during drying, thereby facilitating the observed redispersibility.

#### 3.2.2 Surface properties

To gain a better understanding of the material interaction with water, its wetting properties were compared with contact angles measured from the dried network surfaces. The results are presented in Figure 4.

**FIGURE 4:**
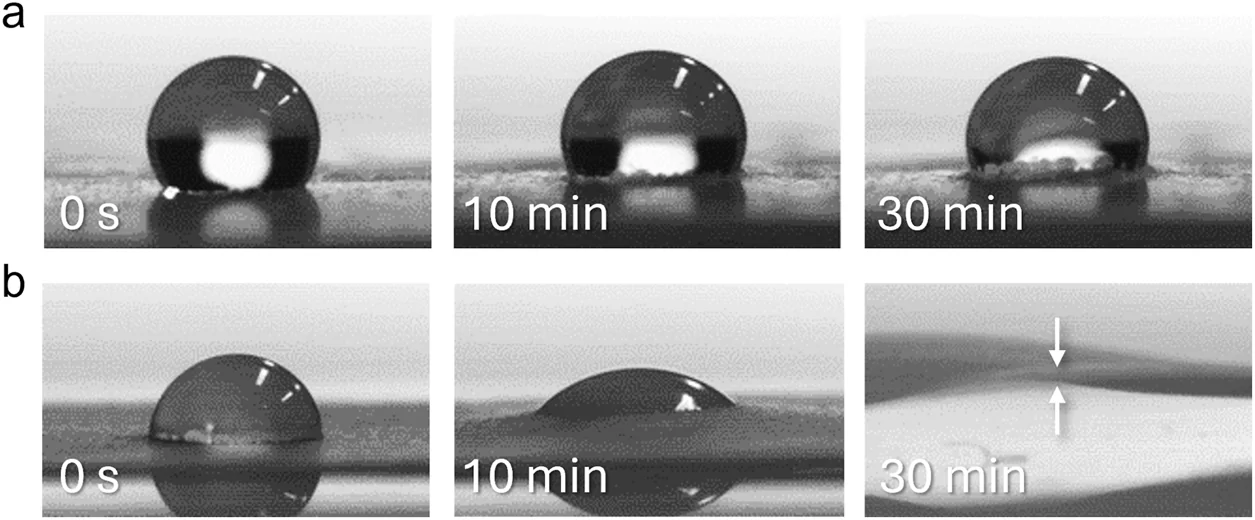
Photographs of water contact angle measurements at instant drop stabilization (0 s), and after 10 min and 30 min of **(a)** cacao nanofiber and **(b)** reference nanofiber dried network, respectively.

From Figure 4, the water contact angles measured from the dried network surfaces after immediate stabilization (corresponding to 0 s) were approximately 120° ± 2 and 81° ± 1 for the cacao (Figure 4a) and reference nanofibers (Figure 4b), respectively. The contact angles measured for cacao nanofibers were relatively stable over time, reaching 99° ± 2 after 30 min, whereas those of the reference nanofibers decreased over time. After 30 min, the drop was completely absorbed, and the curved network remained, as shown in Figure 4b (network indicated by arrows). For food product packaging or coatings, it has previously been reported that cellulosic fiber-based solutions can benefit environmental performance compared to fossil-based counterparts and that the barrier properties are a crucial function (Schenker et al., 2021).

The cacao nanofiber network exhibited an immediately more water-repellent surface, as evidenced by a significantly higher initial water contact angle than the reference nanofibers (Supplementary Table S7). In contrast, the reference nanofibers displayed a more hydrophilic surface with a substantially lower initial water contact angle. This difference in wetting behavior persisted throughout the measurement period, with the cacao nanofibers consistently retaining higher contact angles, whereas the water droplet on the reference nanofibers was rapidly absorbed, preventing meaningful statistical comparison at later time points. The observed difference in wettability between cacao and reference nanofiber networks may be influenced by differences in their chemical composition, including the presence of retained non-cellulosic components such as lipids.

The fatty acids in cacao nanofibers are lipids that contain long-chain hydrocarbons terminated with a carboxylic acid functional group. Because of the long hydrocarbon chain, fatty acids are hydrophobic (water repellent), compared to the hydroxyl group-rich cellulose of the reference nanofibers (Namazi and Dadkhah, 2010). However, wettability is a complex surface phenomenon that is also affected by factors such as surface morphology, roughness, and porosity (He et al., 2023). Therefore, while the retained lipids may contribute to the increased water contact angle of the cacao nanofiber networks, the present results do not allow the hydrophobic behavior to be attributed exclusively to lipids. Additional studies involving surface chemical analysis and/or selective removal of specific components would be required to elucidate the relative contributions of chemical composition and surface topography to the observed wettability behavior. Nevertheless, the comparatively high water contact angles and prolonged resistance to water absorption suggest that cacao nanofibers are of interest for further exploration in functional coating applications.

#### 3.2.3 Bioactivity

Raw cacao is known to be rich in bioactive compounds, and extracts from cacao husk have displayed antioxidant- and antimicrobial activity (Utami et al., 2021). The bioactivity of the nanofibers was characterized and presented in Figure 5. The antioxidant activity of cacao husk is attributed to specific flavonoids and phenolics such as kaempferol, resveratrol, and protocatechuic acid, condensed tannins, theobromine and methylxanthines, and fiber-bound pectin containing phenolic acids such as gallic acid and protocatechuic acid (Campos-Vega et al., 2018; Hernández-Hernández et al., 2019; Dewi et al., 2025). These compounds may be free or loosely bound and are highly extractable.

**FIGURE 5:**
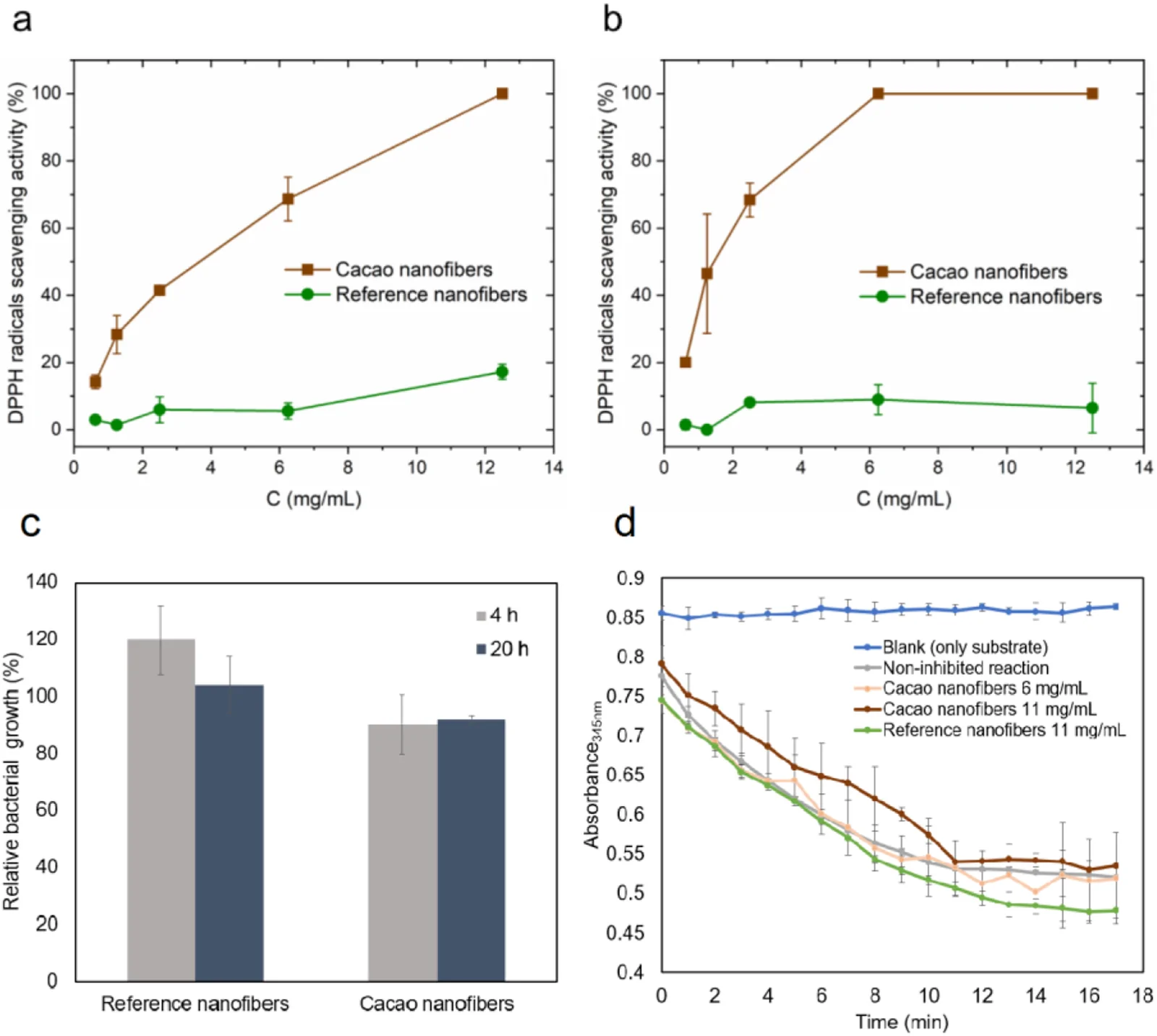
Antioxidant activity of freeze-dried samples prepared from cacao nanofibers and reference nanofibers as a function of the 2,2-diphenyl-1-picrylhydrazyl (DPPH) free radical after **(a)** 30 min and **(b)** 20 h of incubation. **(c)** Antibacterial activity of samples prepared from cacao nanofibers and reference nanofiber gel against *E. coli* (25% v/v in culture medium). **(d)** Anti-collagenase activity of freeze-dried samples prepared from cacao nanofibers and reference nanofibers.

Studies on cacao pod husk have focused on extracting antioxidant compounds (e.g., with ethanol, methanol, and ethyl acetate), rather than evaluating the antioxidant activity of the intact or pretreated fibers themselves. In other words, antioxidant testing, such as using the DPPH assay, is almost always performed on extracts, even though the fiber may contain phenolics and tannins bound within the fiber matrix in the raw husk or after pretreatment. The IC_50_ value of cacao husk extracts, assessed by the DPPH assay, may vary over a range of 5–50 μg/mL, depending on the extraction solvent and methodology (Senthil Amudhan and Apshara, 2015; Umri et al., 2019; El-Lateef et al., 2024). There is only one report on the antioxidant activity of cacao husk fibers, in which 85% of the DPPH radical was inhibited from an untreated cacao pod husk product, exhibiting an IC_50_ value equal to 25 g/g DPPH (Yapo et al., 2013).

From Figures 5a,b, cacao nanofibers showed a promising antioxidant potential because 50% of the free radical was scavenged at 4.4 mg/mL, corresponding to 111 g/g DPPH. A 100% scavenging effect was observed at 12.5 mg/mL solid, after 30 min of incubation (Figure 5a). After 20 h of incubation, a 50% scavenging of the free radical was observed at only 1.7 mg/mL, while a 100% scavenging was shown at 6.3 mg/mL (Figure 5b). The reference nanofibers were assessed as a negative control, and they had negligible antioxidant activity even at the highest tested nanofiber load (12.5 mg/mL). Compared to the previously antioxidant performance of raw cacao husk (IC_50_ 25 g/g DPPH; Yapo et al., 2013), the retained activity of the cacao nanofibers in our study is significant (111 g/g DPPH), especially if the severity of pretreatment and the final macroscopic properties of the nanofibers are taken into account, including the white color and cellulose-like texture, that would not otherwise indicate presence of phenolics (Figure 1). Impressively, the IC_50_ value of the cacao nanofibers (4.4 mg/mL) is within the same range of magnitude as nanofibers produced from biologically pretreated wood from spent mushroom substrate with a 100% scavenging effect at a range of 0.5–2.5 mg/mL (Zhao et al., 2024). The macroscopic properties of these nanofibers, including the intense brown color, are dominated by the presence of lignin and free phenolics.

A leaching test was conducted to understand the mode of binding of antioxidants on the cacao nanofibers. The test was performed at mildly acidic or alkaline pH to cover a wide range of conditions for leaching, depending on the potential application of the nanofiber. It was concluded that there was no leaching of phenolics or compounds with antioxidant activity from the nanofiber within 14 days of incubation. This strongly suggests that the compounds remaining on the nanofiber and contributing to its antioxidant activity are not freely bound or further extractable but are most likely tightly bound within pectin, which is retained at approximately 3% (Table 1). Most of the antioxidant potential in cacao husk comes from polyphenolic compounds, but the lipid fraction, particularly unsaturated fatty acids and phytosterols, may also play a secondary but notable role (Aziz et al., 2021).

The antibacterial activity of cacao husk is generally attributed to extractable phenolic compounds (phenolic acids, flavonoids, and tannins) and phytosterols that are likely to act via oxidative or membrane-targeting mechanisms (Utami et al., 2021; Arbia et al., 2021). Pectin extracted from cocoa pod husk, via citric acid and ethanol, does show antimicrobial activity against *E. coli* and *Staphylococcus aureus.* However, this activity is tied to the extractable fraction, not the fiber itself (Belwal et al., 2022). As shown in Figure 5c, the *E. coli* biomass growth was not inhibited in the presence of reference material, while the cacao nanofibers slightly inhibited the growth by 10%. The trend of inhibition by the cacao nanofibers was maintained at 10% when assessed after 4 h and 20 h of culture (Figure 5c). The formulation at 5% v/v or 10% v/v did not inhibit bacterial growth under the tested conditions. Because the nanofibers lack leachable phenolic or antioxidant compounds, it is hypothesized that the observed, albeit low, mild antibacterial activity is due to the presence of pectin and/or certain fats.

Other bioactivities evaluated were resistance to fiber degradation by lysozyme and collagenase inhibition. Resistance to lysozyme degradation is important for cosmetic or pharmaceutical dressings, such as skin patches, masks, or bioactive wound-covering films, because it enhances their stability when in contact with skin. Products such as sheet masks, wound dressing films, under-eye patches, or transdermal delivery materials must remain intact on the skin for a defined period (e.g., 15 min to several hours). Resistance to lysozyme ensures the material does not prematurely degrade, dissolve, or lose mechanical strength upon skin contact. Lysozyme secreted by the human body plays a key role in the innate immune system, breaking down bacterial cell walls by degrading certain polysaccharides (e.g., chitin, peptidoglycan, and some glycosidic bonds in biomaterials) (Zhao et al., 2023). The cacao nanofibers were not degraded in the presence of lysozyme when incubated at pH 5.8 or 7.4 for 14 consecutive days. The nanofiber is resistant to lysozyme, as its polysaccharides do not contain the enzyme’s substrate (peptidoglycan with β(1→4) bonds between N-acetylmuramic acid and N-acetylglucosamine).

Collagenase is a type of matrix metalloproteinase that breaks down collagen, the main structural protein in skin and connective tissue. The body produces collagenase during wound healing or inflammation, but excessive or uncontrolled collagenase activity can be damaging (Gill and Parks, 2018; Caley et al., 2015). Collagenase is a natural and essential enzyme in the wound healing process; thus, its function should not be inhibited during the early or middle stages of healing. During this process, it helps remove damaged collagen and necrotic tissue, remodel the extracellular matrix to allow new cell migration, and facilitate angiogenesis and granulation tissue formation (Han and Ceilley, 2017; Schultz et al., 2011). In chronic wounds, collagenase and other matrix metalloproteinases are often overexpressed, leading to degradation of newly formed collagen, breakdown of granulation tissue, delayed healing, and increased risk of chronic inflammation and scarring. As such, it is not necessary to block collagenase entirely, but moderating its activity, especially in chronic or poorly healing wounds, is often essential for effective tissue repair and regeneration (Liu et al., 2009). Therefore, when a dressing is intended to protect a wound and support the initial stages of healing, collagenase inhibition is generally not desirable. However, if the dressing is applied in later healing stages, a moderate inhibition of the collagenase activity may be beneficial to support tissue remodeling and prevent excessive matrix degradation.

Anti-collagenase activity is also desired in anti-aging products, such as serums, creams, sheet masks, and functional skincare formulations, to preserve collagen (types I and III), which is critical for skin strength, firmness, and elasticity (Quan et al., 2009). The cacao nanofibers produced in this study reduced the initial rate of collagenase by 16.7% at 11 mg/mL, indicating partial inhibition and suggesting that the nanofibers have moderate efficacy under the tested conditions (Figure 5d). At cacao nanofiber concentrations ranging from 0.5 mg/mL to 6.0 mg/mL, no anti-collagenase activity was observed. For reference nanofibers, no anti-collagenase activity was detected at the tested concentrations. The acidified ethanol extract of cacao pod husk has previously shown an anti-collagenase IC_50_ of 0.43 mg/mL, alongside other activities such as antioxidant, anti-tyrosinase, and anti-glycation effects (Anatachodwanit et al., 2025). This demonstrates that bioactive polyphenols from cacao husk can inhibit collagenase enzyme activity, which is beneficial for skin aging prevention and scar formation. However, this property was not strongly preserved in the presently produced nanofiber from cacao residue.

Thus, cacao nanofibers have potential for further development as a versatile material, for example, as wound dressings with potential use during both the early and later stages of healing. However, several limitations must be addressed before biomedical applications can be considered. The present study did not include cytotoxicity evaluation; the biological safety of the materials remains to be established. As cytocompatibility is influenced by multiple factors, including the raw material source, processing conditions, sterility, and residual chemicals, further studies on skin cell cytotoxicity, cell migration, and *in vitro* and *in vivo* biocompatibility are required before cacao nanofibers can be safely proposed as functional wound dressing materials.

## 4 Conclusion

After pretreatment of cacao residue, the resulting composition primarily consisted of carbohydrates: cellulose, hemicellulose, and pectin (30 wt.%) and extractives (50 wt.%), mainly non-polar extractives that consisted of fat. Preservation of non-cellulosic components resulted in a high yield of 30 wt.% and an energy demand upon fibrillation corresponding to 3.9 kWh/kg, compared to 8.2 kWh/kg for the reference material. The rich composition, including high fat content of cacao nanofibers, resulted in specific multifunctional properties combining excellent redispersibility, hydrophobic nature, and bioactivity, which were not observed for the reference material. However, the high fat content influenced the network formation ability, as displayed in a lower viscosity and mechanical properties of the prepared dried networks compared to the reference material. This study demonstrates that preserving the intrinsic composition of cacao residue provides a resource-efficient strategy for producing multifunctional nanofibers with potential for further development toward value-added applications, including coatings, cosmeceuticals, and biomedical materials.

## Data Availability

The raw data supporting the conclusions of this article will be made available by the authors, without undue reservation.
