# Supplementary material for: Resource-efficient use of industrial residue for multifunctional cacao nanofibers
Source: Front Bioeng Biotechnol. 2026 Oct 5;14:1916485. doi: 10.3389/fbioe.2026.1916485 (PMC13677836; doi:10.3389/fbioe.2026.1916485)
Supplement: Supplementary file 1 [file DataSheet1.pdf]

Supplementary Material

Supplementary Table 1. Composition of hemicelluloses for cacao residue before and after pretreatment in (w/w), standard deviations are shown in brackets.

	Arabinan	Galactan	Mannan	Xylan
Cacao residue	0.9 (0.0)	1.3 (0.0)	0.8 (0.1)	0.6 (0.0)
Pre-treated	0.8 (0.0)	1.7 (0.0)	1.7 (0.0)	2.6 (0.0)

Supplementary Table 2. Fatty acid composition of cacao samples (expressed as % of total fatty acids).

	**Cacao residue**	**Pretreated**
Esterified fatty acids		
Stearic acid (C18:0)	37.3	1.3
Oleic acid (C18:1)	34.2	42.2
Palmitic acid (C16:0)	17.8	22.9
Linoleic acid (C16:2)	6.1	28.6
Arachidic acid (C20:0)	2.3	3.1
Free fatty acids		
Oleic acid	30.5	12.8
Palmitic acid	26.4	47.1
Stearic acid	24.3	35.8
Linoleic acid	15.5	< 0.1
**Arachidic acid**	**< 0.1**	**< 0.1**

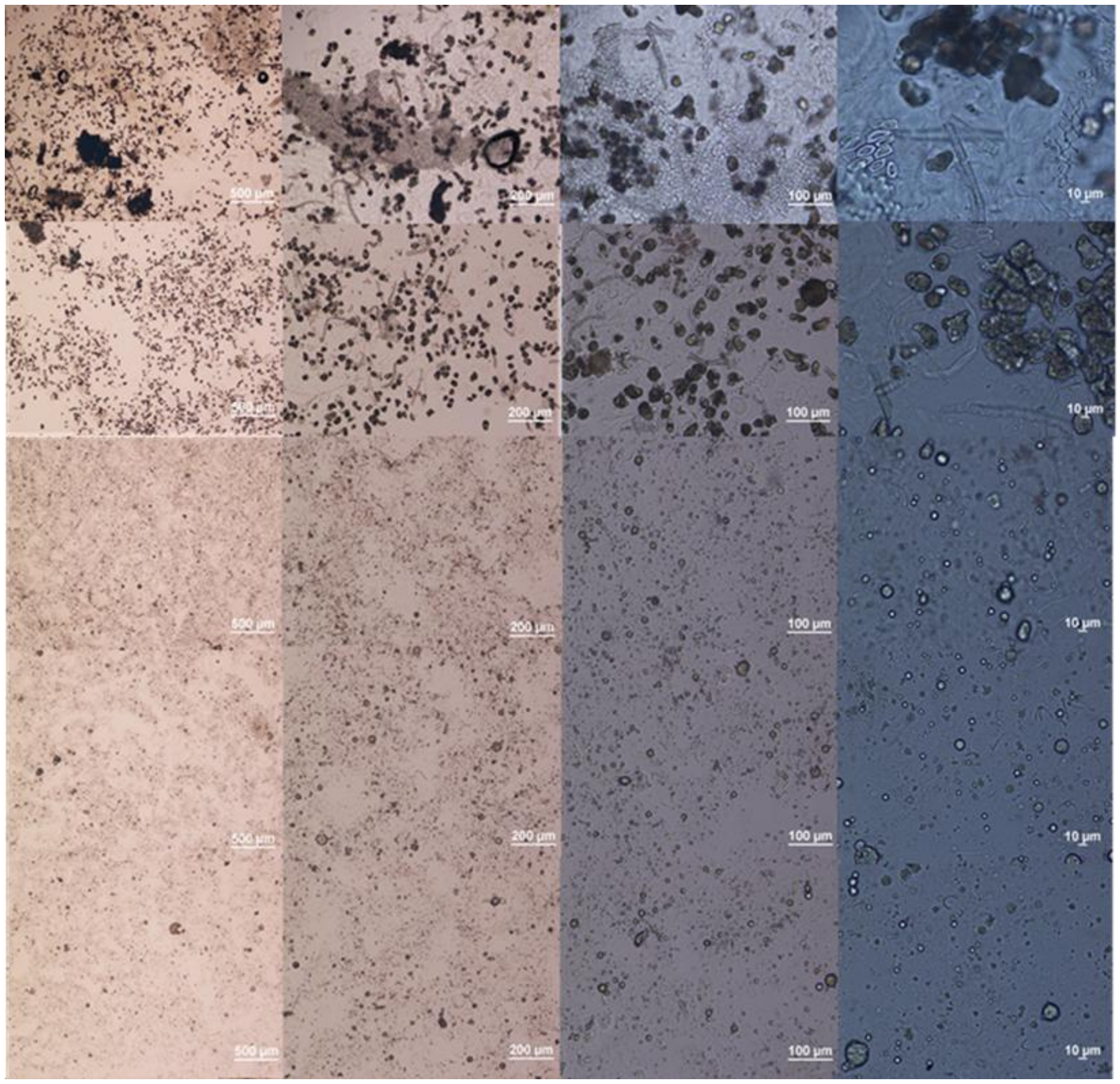

Supplementary Figure 1. OM images at different magnifications captured during the fibrillation process into cacao nanofibers.

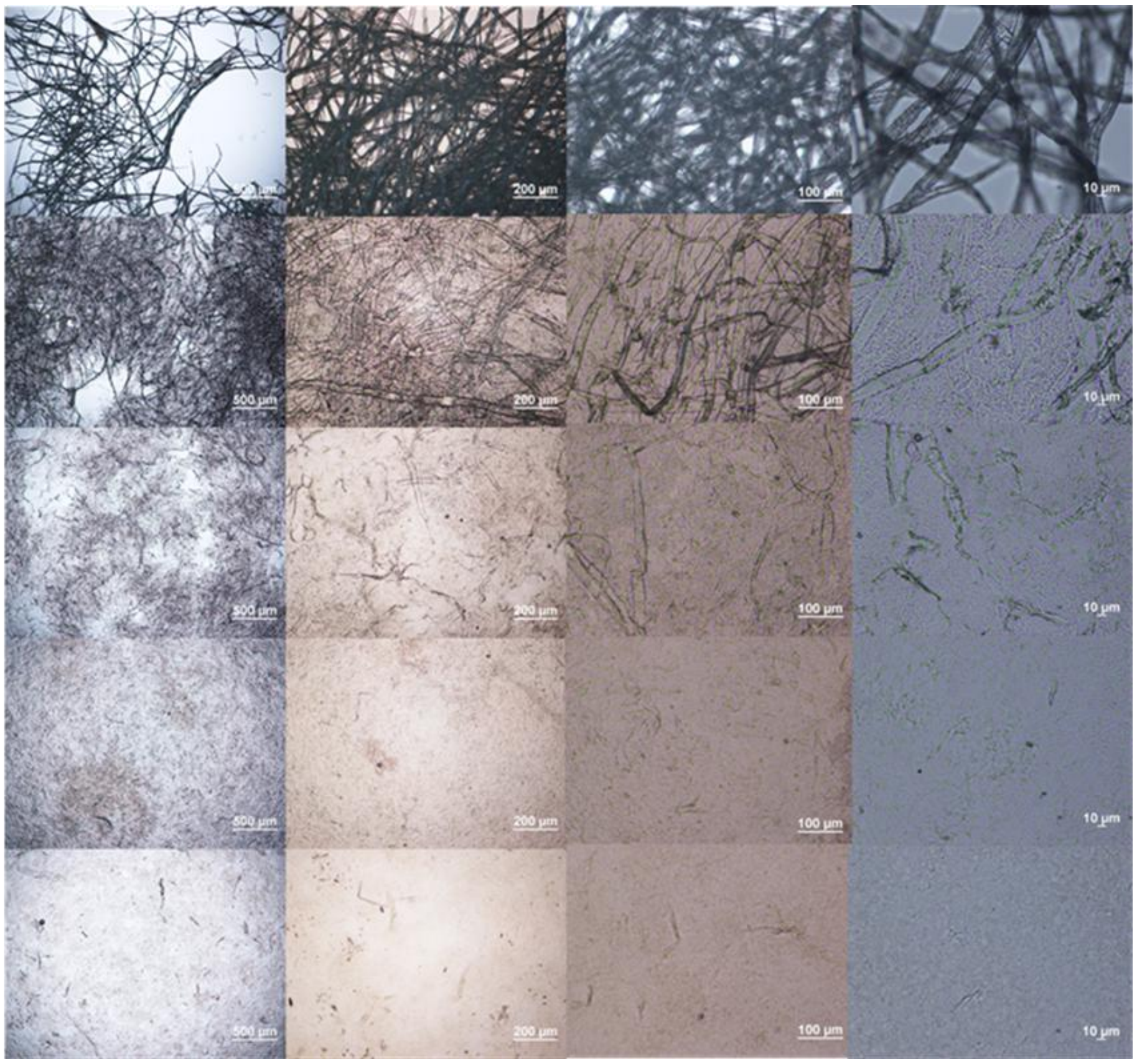

Supplementary Figure 2. OM images at different magnifications captured during the fibrillation process into reference nanofibers.

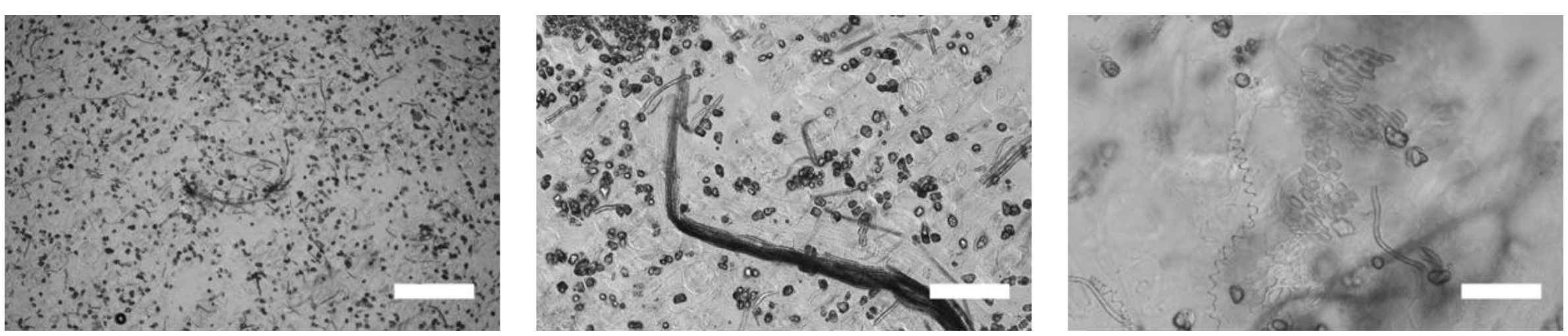

Supplementary Figure 3. OM images at different magnification 500µm, 200 µm, 100 µm (from left to right) of the pretreated cacao residue.

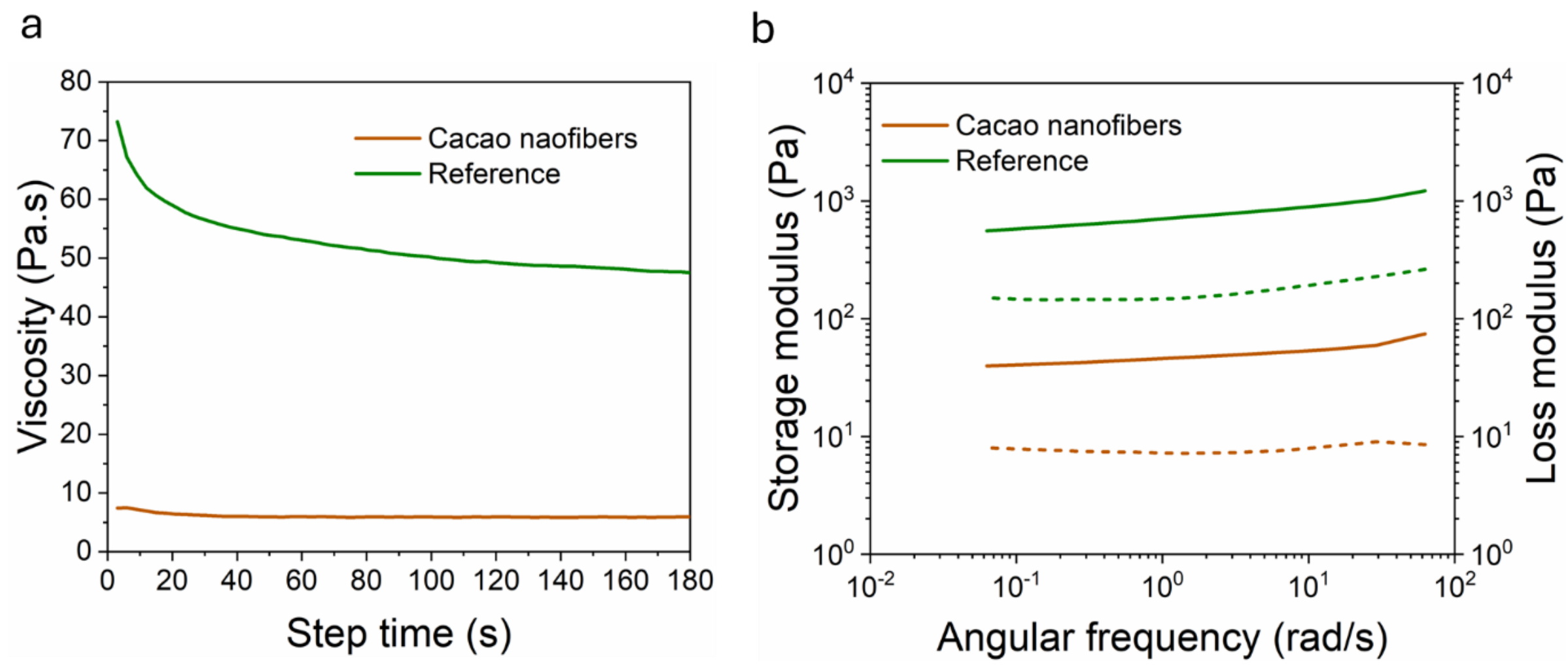

Supplementary Figure 4. (a) Flow peak hold with 1 s^{-1} shear rate was used and maintained for 180 s and (b) Oscillatory frequency sweep at a strain of 1%, frequency variation from 0.01 to 10 Hz for cacao- and reference nanofibers, respectively.

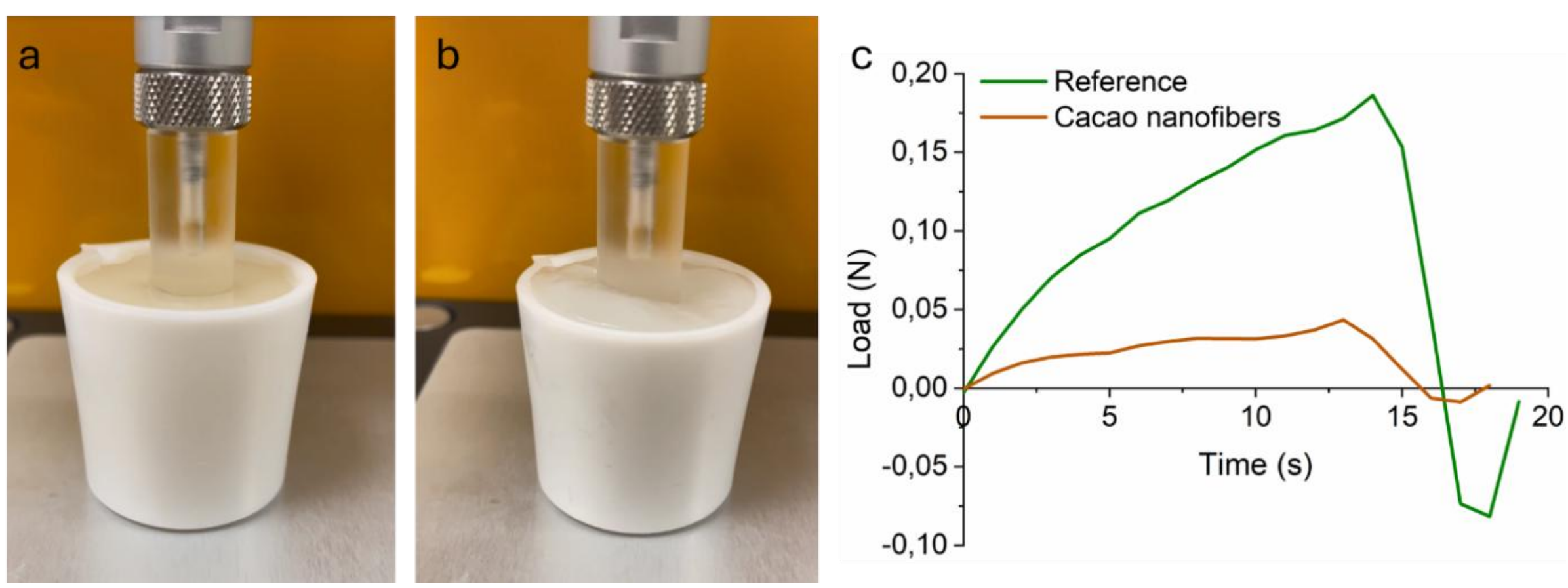

Supplementary Figure 5. Photos from texture analyzer testing (a) cacao nanofibers and (b) reference nanofibers. (c) Load as a function of time for cacao- and reference nanofibers, respectively.

Supplementary Table 3. Values from texture analyzer (Figure S5c) for cacao- and reference nanofibers.

**Parameter**	**Cacao nanofibers**	**Reference**
Peak load (hardness)	0.042 N	0.175 N
Work (total positive area)	0.39 mJ	1.86 mJ
Adhesion (total negative area)	-0.009 mJ	-0.14 mJ
Adhesive force	-0.007 N	-0.096 N

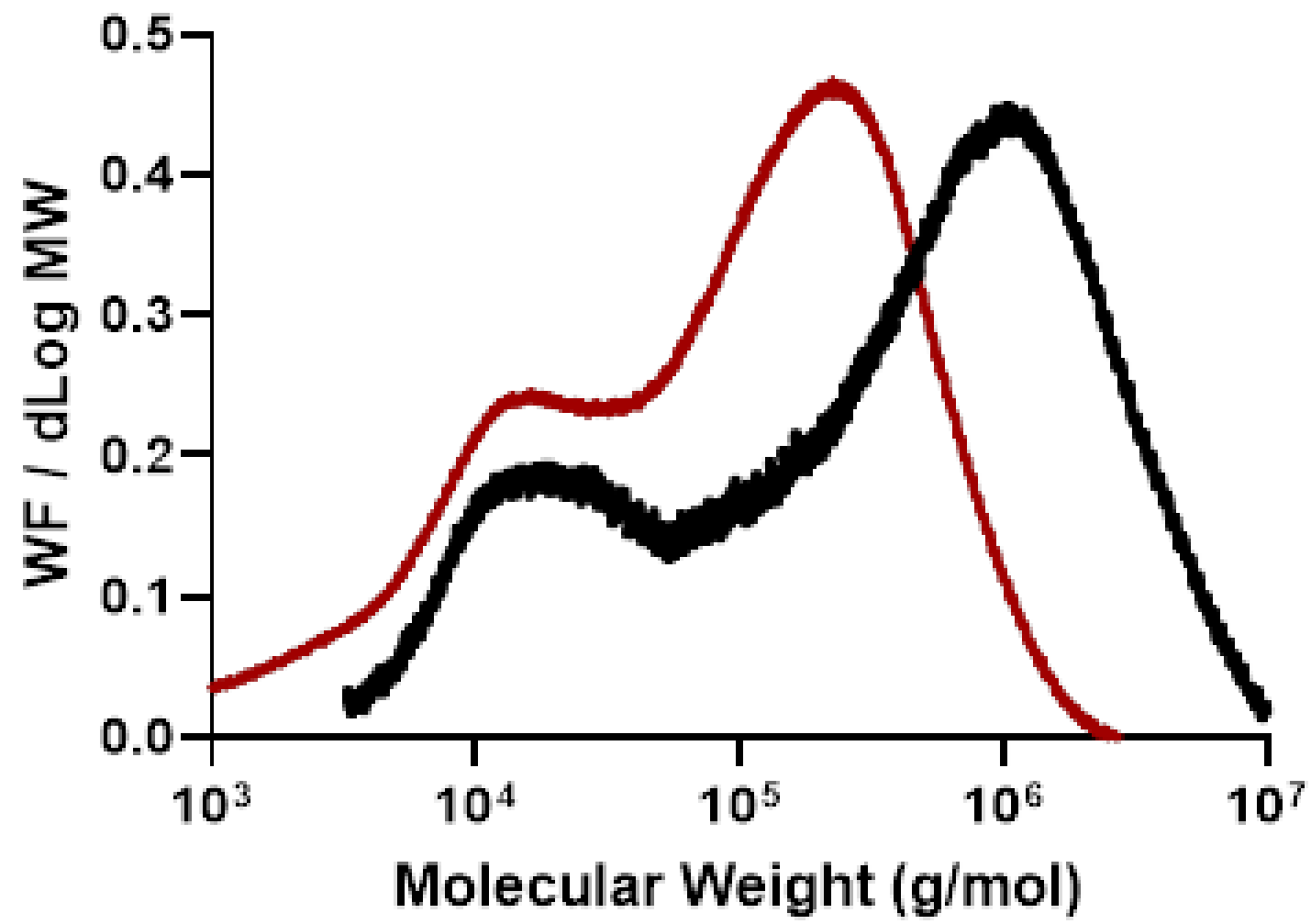

Supplementary Figure 6. The relative molecular weight distribution of the pretreated cacao residue black line, and the cacao nanofibers, red line, compared to Pullulan standards.

Supplementary Table 4. Numerical summary of the molecular weight distribution, in g/mol, where M_w is the weight average molecular weight, M_n is the number average molecular weight, and PDI is the polydispersity index (M_w/M_n).

**Material**	**M_n (STDEV %)**	**PDI (=M_w/M_n)**
Pretreated cacao residue	45912 (4)	18
Cacao nanofibers	17355 (7)	11

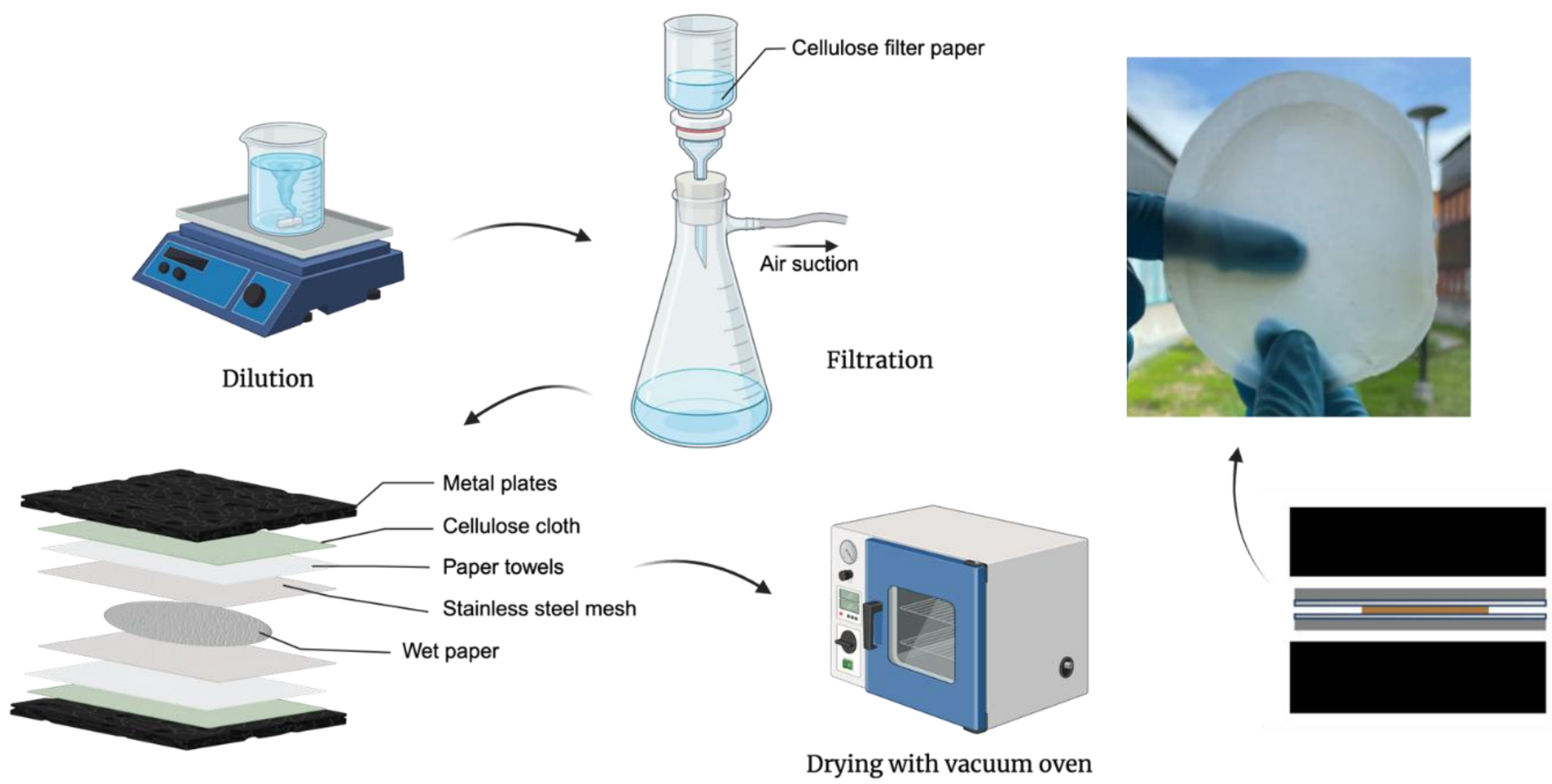

Supplementary Figure 7. Preparation process of dried networks from cacao- and reference nanofibers for the redispersion study.

Supplementary Table 5. Viscosity of cacao- and reference nanofiber gels measured before and after redispersion. Statistical analysis of the difference of viscosity evaluated using Welch's independent-samples t-test. Statistical significance was accepted at $p < 0.05$. Values are presented as mean ± standard deviation (n = 3 per material).

**Material**	**Before** redispersion	**After** redispersion	**Welch's *t***	***p*-value**	**Interpretation**
**Cacao nanofibers**	351 ± 5	427 ± 36	3.6	0.066	No significant change (increasing trend)
**Reference**	1610 ± 24	696 ± 37	28.8	<0.001	Significant decrease

Supplementary Table 6. Effect of redispersion on the mechanical properties of the dried networks from cacao- and reference nanofibers tested in tensile mode before and after redispersion. Statistical analysis of the difference of mechanical properties evaluated using Welch's independent-samples t-test. Statistical significance was accepted at $p < 0.05$. Values are presented as mean ± standard deviation (n = 10 per material).

**Material**	**Property**	**Before** redispersion	**After** redispersion	**Welch's *t***	***p*-value**	**Interpretation**
**Cacao nanofibers**	Tensile strength (MPa)	22.3 ± 2.4	24.7 ± 2.8	2.1	0.048	Significant increase
	Strain at break (%)	2.6 ± 0.4	3.4 ± 0.3	5.3	<0.001	Significant increase
	Elastic modulus (GPa)	2.3 ± 0.3	2.1 ± 0.6	0.7	0.474	No significant change
**Reference**	Tensile strength (MPa)	115.7 ± 6.1	105.2 ± 10.7	2.7	0.017	Significant decrease
	Strain at break (%)	6.7 ± 0.7	4.3 ± 0.6	8.9	<0.001	Significant decrease
	Elastic modulus (GPa)	5.5 ± 0.5	5.1 ± 0.8	1.3	0.2	No significant change

Supplementary Table 7. Water contact angle measurements of cacao- and reference nanofiber network surfaces. Statistical analysis of the difference of instant water contact angles evaluated using Welch's independent-samples t-test. Statistical significance was accepted at $p < 0.05$. Values are presented as mean ± standard deviation (n = 10 per material).

**Material**	**Water contact angle after 0s (°)**	**Welch's *t***	***p*-value**
**Cacao nanofibers**	120 ± 2	66.8	<0.001
**Reference**	81 ± 1		
